# Assessment of scientific thinking in basic science in the Iranian second national Olympiad

**DOI:** 10.1186/1756-0500-5-61

**Published:** 2012-01-23

**Authors:** Negar Azarpira, Mitra Amini, Javad Kojuri, Parvin Pasalar, Masud Soleimani, Saman Hossein Khani, Marzieh Ebrahimi, Hassan Niknejhad, Zahra Karimian, Farhad Lotfi, Shahram Shahabi, Iraj Saadat, Mohammad Reza Dehghani, Mohammad Ali Mohagheghi, Payman Adibi, Kamran Bagheri Lankarani

**Affiliations:** 1 Transplant Research Center, Shiraz University of Medical Sciences, Shiraz, Iran; 2 Education Development and Research Center, Shiraz University of Medical Sciences, Shiraz, Iran; 3 Department of Biochemistry, Tehran University of Medical Sciences, Tehran, Iran; 4 Tarbiat Modares University, Tehran, Iran; 5 Department of Regenerative medicine, Royan Institute for stem cell biology and technology, ACER, Tehran, Iran; 6 Nanomedicine and Tissue Engineering Research Center, Shahid Beheshti University of Medical Sciences, Tehran, Iran; 7 Department of Immunology, Uramieh University of Medical Sciences, Uramieh, Iran; 8 Department of Biology, College of Sciences, Shiraz University, Shiraz, Iran; 9 Vice Chancellor for Educational Affairs, Ministry of Health and Medical Education, Tehran, Iran; 10 Integrative Gastroenterology Research Center, Department of internal medicine, Isfahan University of medical sciences, Isfahan ,Iran; 11 Health Policy Research Center, Shiraz University of Medical Sciences, Shiraz, Iran

## Abstract

**Background:**

To evaluate the scientific reasoning in basic science among undergraduate medical students, we established the National Medical Science Olympiad in Iran. In this Olympiad, the drawing of a concept map was used to evaluate a student's knowledge framework; students' ability in hypothesis generation and testing were also evaluated in four different steps. All medical students were invited to participate in this program. Finally, 133 undergraduate medical students with average grades ≥ 16/20 from 45 different medical schools in Iran were selected. The program took the form of four exams: drawing a concept map (Exam I), hypothesis generation (Exam II), choosing variables based on the hypothesis (Exam III), measuring scientific thought (Exam IV). The examinees were asked to complete all examination items in their own time without using textbooks, websites, or personal consultations. Data were presented as mean ± SE of each parameter. The correlation coefficient between students' scores in each exam with the total final score and average grade was calculated using the Spearman test.

**Results:**

Out of a possible score of 200, the mean ± SE of each exam were as follows: 183.88 ± 5.590 for Exam I; 78.68 ± 9.168 for Exam II; 92.04 ± 2.503 for exam III; 106.13 ± 2.345 for Exam IV. The correlation of each exam score with the total final score was calculated, and there was a significant correlation between them (*p *< 0.001). The scatter plot of the data showed a linear correlation between the score for each exam and the total final score. This meant that students with a higher final score were able to perform better in each exam through having drawn up a meaningful concept map.

The average grade was significantly correlated with the total final score (R = 0.770), (*p *< 0.001). There was also a significant correlation between each exam score and the average grade (*p *< 0.001). The highest correlation was observed between Exam I (*R *= 0.7708) and the average grade. This means students with higher average grades had better grades in each exam, especially in drawing the concept map.

**Conclusions:**

We hope that this competition will encourage medical schools to integrate theory and practice, analyze data, and read research articles. Our findings relate to a selected population, and our data may not be applicable to all medical students. Therefore, further studies are required to validate our results.

## Background

Science is an activity that consists of the explanation, prediction, and control of empirical phenomena in a rational manner. By "scientific reasoning," we mean the rules of reasoning relevant to carry out this activity, including principles governing experimental design, hypothesis testing, and data interpretation. The development of expertise is not only the result of acquiring more knowledge and skills; structuring knowledge is also a critical step. Thus, integrated frameworks of related concepts, which facilitate problem solving and other cognitive activities, are very important. Scientists must master two skills: recognizing where to look and knowing what is seen. The first ability is achieved by experimental design, and the second ability is hypothesis generation, which involves theory evaluation. The traditional method of assessment involves measuring information; analysis and organization of new knowledge and critical thinking with meaningful learning are often omitted [1-4].

There are many science competitions around the world that provide encourage students to learn about science. Many of them are knowledge-based while others focus mainly on the students presenting a research project and discussing their findings.

The aim of International Olympiads, such as in physics, chemistry, and biology, is not only to encourage excellence through competition, but also to enhance international goodwill among nations through personal contacts and increased understanding of the diversity of cultures and traditions. They also demonstrate the role of teamwork in solving scientific problems, stimulate interactions between students and teachers, and may help improve science education at national and international levels [5].

As an undergraduate medical student develops into a skilled specialist or PhD student, one important step has to be made, which is to bridge the gap between textbook knowledge and scientific skills. From textbooks, medical students can gather knowledge in the form of review articles, which represent the general consensus of the scientific community; however, with many research articles presented in journals, consensus about observations and interpretations has generally not yet been reached. It is important to engage students actively in developing their knowledge, and it is necessary for them to focus on concepts applicable to solving problems and relating previous knowledge to new knowledge [6,7].

Concept mapping is a tool that can represent knowledge structure by illustrating the relationships between relevant concepts within a given subject domain. By relating and integrating new knowledge with an existing knowledge structure, students develop a deeper understanding, allowing better use of knowledge to generate hypotheses, design experiments, and test the variables to find the answers to scientific questions [8,9].

The Iranian National Medical Science Olympiad was designed to encourage undergraduate medical students to bridge the gap between textbook learning and scientific reasoning. In recent years, stem cell research has been grown exponentially, and stem cell-based therapies have the potential to improve the life of patients with such conditions as Parkinson disease, myocardial infarction, and diabetes mellitus [10,11]. Therefore, stem cell differentiation was chosen as the subject of the present study. In the National Medical Science Olympiad, the drawing up of a concept map was used to measure aspects of students' knowledge framework, and their ability to generate and test hypotheses was also evaluated. The correlation between the students' scores in each step and their final total score was further investigated.

## Methods

The design of the National Medical Science Olympiad in Iran is focused on critical thinking among medical students. The first Olympiad was held in Isfahan in 2009, and the second in Shiraz in 2010 [12,13]. The specific goals of the Olympiad were competition to achieve the following: identifying and encouraging scientifically talented students; generating scientific morale; helping students gain closer familiarity with scientific culture; cultural exchanges among students; encouraging teamwork to develop creative and critical thinking; reinforcing the goals and objectives of the health system; further the development of interdisciplinary activities [12].

The Ethics Committee of the Olympiad approved the protocol adopted in the present study, and written, informed consent was obtained from all participants. Currently enrolled medical students who were interested in the study topic and who had an average grade of 16/20 (equivalent to a GPA of about 3.2 in the United States or a class of about 60 in the United Kingdom) or higher were able to register for the study. They then took part in an intensive training course in the subject area of their choice at their own university. Enrollees were tested, and those with the highest grades were allowed to participate in the study.

Iran has 46 medical universities, and each university is allowed to send only three students in the field of basic science to the Olympiad. A total of 133 undergraduate students took the test in basic science. In this study, we analyzed the examination results in basic science.

### Development of the Olympiad examination by the reference panel

To prepare the examination used in the Olympiad, 10 experts from different universities in Iran were chosen for the reference panel. These experts held PhDs in such areas of basic science as biochemistry, hematology, physiology, immunology, pharmacology, and pathology, though they had different levels of professional experience. Each member of the panel took each of the four tests. On the morning of the first day, the drawing up of a concept map was completed; in the afternoon, the hypothesis writing was completed. On the morning of the second day, the hypothesis testing was evaluated; in the afternoon, the measuring of scientific thought was investigated. Each of the four examination periods lasted 4 h.

### Examinees

All the medical students were invited to participate in this program. The selected examinees were 133 undergraduate medical students from 45 medical schools in Iran who had grades ≥ 16/20. The length of medical education in Iran is 7 years. Forty-three percent of the participants were male, and 57% were female. The mean age of the participants was 21.3 years, and their mean average grades were 18.3/20. The examinees were asked to complete all the Olympiad examination items in their own time without using textbooks, websites, or personal consultations.

### Exam I: drawing a concept map

On the morning of the first day, the students were asked to draw a concept map based on three new articles in the field of stem cell research [14-16]. The examination period lasted 4 h.

To learn how to make concept maps, each participant previously completed a standardized concept map training session. Briefly, training included an introduction to concept mapping, followed by practice making concept maps on medical topics. The raters also received training about the concept-mapping process in the National Medical Science Olympiad.

Participants were given 90 min to complete their maps. The maps were coded, so that the identity, specialty, and level of training of the participants would be unknown to the raters. Participants created the maps on 20 × 30-cm sheets of paper. In constructing their concept maps, the participants drew concepts related to a certain domain and then indicated links using arrowed lines with a proposition written above the line, describing how the concepts were related (concept link), [8,9]. Map hierarchy is an important part of the process; it is indicated by the direction of the arrow in the concept link and in the arrangement of concepts in the map with more general concepts at the top and more specific concepts below. Scoring of each concept map was based on the following criteria, using previously published reports [8,9]:

1) Valid and meaningful selection of concepts from the papers (score, 25%).

2) Hierarchical arrangement of concepts with more general concepts at the top and more specific ones below (score, 20%).

3) Meaningful integration among concepts in the map (score, 10%). Incorrect concept relationships were given zero points. A closely related concept was given the highest number of points. Less important, but correct concept relationships were given an intermediate score.

4) Accuracy and depth of understanding of the relationship (score, 20%).

5) Degree of student creativity showing more sophisticated understanding (score, 25%).

Propositions or cross-links that lacked linking phrases above the connecting line were counted separately and given less credit. No credit was given for an invalid (i.e., incorrect or wrong) proposition or cross-link. Before scoring the maps, the raters discussed problems with each other. Raters scored a random subset (40%) of the same maps to assess reliability. Three different raters, with experience in stem cell field research and blinded to the identity of the map author, independently scored each map. The raters' total scores were added to create a final score for each map. The use of raters who were familiar with both the Olympiad process and the assessment of the different steps involved in making concept maps was believed to be essential in obtaining low inter-rater variability.

### Exam II: hypothesis generation

**Figure 1:**
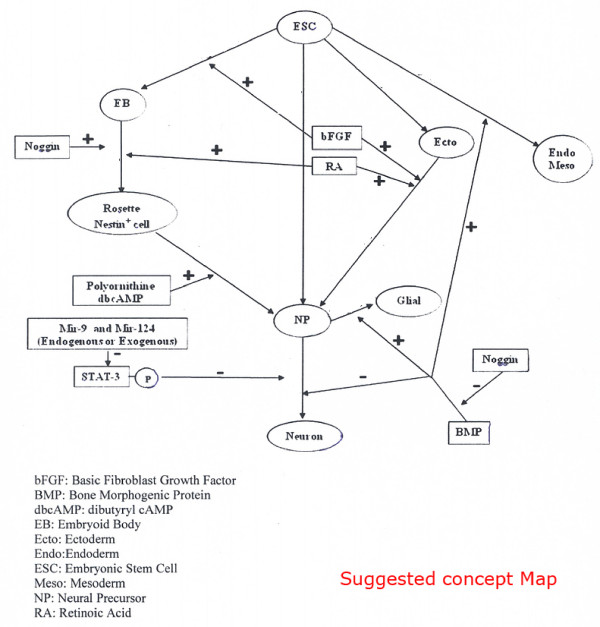
**Suggested Concept Map**.

In the afternoon of the first day, the measuring of scientific thoughts was investigated in terms of hypothesis generation. After drawing their concept maps, the students were asked to write a hypothesis. A sample of a concept map that was drawn by the scientific committee was also available during the exam period (see Additional file 1 and Figure 1). This exam lasted 1 h.

The written hypothesis was evaluated by these criteria:

1) Simplicity (score, 15%)

2) Accuracy (score, 20%)

3) Precision (score, 10%)

4) Explaining the connection between events (score, 15%)

5) Logical coherence (score, 10%)

6) Fruitfulness (score, 10%)

7) Creativity (score, 20%)

### Exam III: choosing variables based on the hypothesis

On the morning of the second day, the participants were asked to choose variables that were important in hypothesis testing. This step lasted 2 h. It was made clear to the participants that they should choose variables that helped prove the hypothesis they had detailed. The credit given for the best answer was 50%, and scientific explanation was scored up to an additional 50%.

### Exam IV: measuring scientific thought

In the final step, on the afternoon of second day, the students were presented with a scientific finding. To measure the students' scientific thinking, they were asked a series of questions related to the finding. They were also asked to make suggestions for future research. This step also lasted 2 h. The task for the examinees was to evaluate their suggestions in terms of direction (positive, negative, or neutral) and intensity. This effect was captured with a Likert scale. A sample of each examination is given in Additional file 1.

### Total final score

The total exam score was assessed using the sum of the four exam grades, with each exam accounting for 25% of the total score.

## Analysis

The data were entered and analyzed using the Statistical Package for Social Sciences (SPSS) version 17.0 (SPSS, Inc., Chicago, IL, USA). Data were presented as mean ± SE of each parameter. The correlation coefficient between the students' scores in each exam (I to IV) with the total final score and average grade was determined using the Spearman test. A *p*-value of less than 0.05 was considered statistically significant.

## Results

Demographically, the study population was 43% male, 57% female. Out of a possible score of 200, the mean ± SE of each exam were as follows: 183.88 ± 5.590 for Exam I; 78.68 ± 9.168 for Exam II; 92.04 ± 2.503 for Exam III; 106.13 ± 2.345 for Exam IV. The correlation of each exam score with the total final score was calculated (Table 1), and there was a significant correlation between them (*p *< 0.001). This means that students with a better final score were able to perform better in each exam through having drawn a meaningful concept map. The scatter plot of the data showed a linear correlation between the score for each exam and the total final score.

**Table 1:** Correlations between total Olympiad examination score and scores in each of the four scientific thinking tests

Exam IV	Exam III	Exam II	Exam I	Correlation coefficient
0.482	0.487	0.661	0.770	Total grade

< 0.001	< 0.001	< 0.001	< 0.001	Significance (*P *value)

The correlation of each exam score with the medical university average grade was also calculated (Table 2). There was a significant correlation between each exam score and the average grade (*p *< 0.001). The average grade was also significantly correlated with the total final score (R = 0.770), (*p *< 0.001).

**Table 2:** Correlations between average grade and scores in each of the four scientific thinking tests

Exam IV	Exam III	Exam II	Exam I	Correlation coefficient
0.553	0.557	0.628	0.778	Grade point average

< 0.001	< 0.001	< 0.001	< 0.001	Significance (*P *value)

The highest correlation was observed between the result for Exam I (*R *= 0.7708) and average grade. This means that students who had a higher average grade had a better grade in each exam, especially in drawing the concept map.

## Discussion

Learning is not considered to be the transmission of data to passive receivers. It is an active process in which students actively develop their knowledge and map new information upon prior knowledge [6,7]. Therefore, active learning methods, such as problem-based learning, collaborative learning, and experiential forms of learning, manipulate information and ideas in ways that help create new meanings or solve problems. A superficial approach to learning is characterized by establishing a base of scientific facts through lectures and note taking.

Conversely, a deep approach to learning is characterized by focusing on the concepts applicable to solving problems and relating previous knowledge to new knowledge. This approach eventually results in the development of a collaborative learning environment and the use of acquired theories, concepts, and knowledge to solve new problems [6,17].

Heparin, insulin, the sinoatrial node, and ether anesthesia are just some of the discoveries that have been made by medical students. In the latter half of the nineteenth century, European medical students had to write and defend a research thesis to achieve their doctorate in medicine. This was the platform on which the discoveries of Raynaud (discovery of Raynaud's disease or phenomenon), Langerhans (discovery of islet cells in the pancreas, which secrete insulin, and also the dendritic cells in the skin known as Langerhans cells), and Duchesne (discovery of a type of muscular dystrophy) were built [18].

In Great Britain and North America, summer research projects were apparently useful for Flack (discovery of the sinoauricular node and heart pacemaker), McLean (discovery of heparin, an anticoagulant), and Best (discovery of insulin). They were the result of intense effort in integrating theory with practice. To quote the great inventor Thomas Edison (1847-1931), "Success is ten percent inspiration and ninety percent perspiration" [18].

In the modern world, in which different areas of science are rapidly developing, giving direction to medical students is clearly very important. It is necessary to stress the role of basic science in medical education and the development of clinical reasoning skills. Thus, greater incorporation of basic science into the medical student's curriculum would seem to be required [19].

In this way, current knowledge about a specific topic derived from the literature has to be translated into questions and solutions obtained though the practical design of a project. This process requires critical thinking, knowing which methods and technologies are currently being used and developing collaborations with other scientists through oral and written communication. In the present article, we describe our experience in a national competition to bridge the gap between textbooks and scientific thinking among talented medical students. It was our aim to stimulate students to develop research (thinking) skills within a limited time frame.

Drawing a concept map, which is based on the constructivism theory of learning, was used to measure knowledge structure. This study demonstrates that concept map scores have a strong correlation with both average grades and total examination score. Better training and more practice was associated with the ability to draw better maps in terms of organization of knowledge, understanding the relationships between concepts, and greater creativity. The use of concept maps by students as a part of their educational training has previously been studied. McGaghie et al. noted that following a focused intervention and training, students' maps relating to concepts of pulmonary physiology became similar to those drawn by faculty experts [20].

A high correlation between each exam result and the total Olympiad grade was an indicator of the concurrent validity of the tests in the present study and also in the construct validity of the whole examination. As noted above, women constituted 57% of the participants in this study. Lopatto reported that men and women did not differ in terms of research interest or overall plans to continue their education, though other authors differed on this point [21,22].

The influence of the faculty mentor or supervisor on a student's grade and previous research experience were not evaluated in the present study. Russell et al. found little evidence of a relationship between mentor characteristics and student-reported outcomes [23]. However, Lopatto believed that mentoring had a significant effect on undergraduate research experience [24].

The strengths and limitations of the present study should be considered. The participation of a large sample of examinees from all Iranian medical universities is an important strength with this study. In addition, a panel of experts from different medical universities advised the study team in the test selection and adaptation, and we think this is very important in the effort to achieve accurate results.

This study was limited to a strictly screened and select number of the best medical students, and thus our findings may not apply to other institutions. Sampling all possible students who may have volunteered for this study at other medical schools may have produced different results. Therefore, the scores cannot be generalizable to the whole population of medical students. This study lacked the potential for a classic experimental comparison with a control group. The lack of control groups in this type of comparison is a common problem [21].

The report of the Academic Competitiveness Council (U.S. Department of Education, 2007) reviewed studies relating to the success of Science, Technology, Engineering, and Mathematics (STEM) education programs and concluded that only 10 of 115 programs were "scientifically rigorous" by including appropriate control groups [25]. The creation of a proper control, such as a student group that applied but was not selected, is practically difficult. Finally, nationally and privately funded grants encourage institutions to recruit talented medical students to share in research opportunities.

## Conclusions

The results of this study shed light onto medical student engagement in basic science. We hope National Medical Science Olympiad will improve the abilities of medical schools to integrate theory and practice, analyze data, read primary literature and research articles, develop skills in scientific writing, increase their confidence, and work independently. However, further studies are required to validate our assessment and to improve such technical aspects as computerized administration and test scoring.

## Competing interests

The authors declare that they have no competing interests.

## Authors' contributions

NA, MA, JK, PP, MS, SH, ME, SH, ME, HN, SSH, and IS contributed to the conception, design of the exam, and acquisition of data; ZK, FL, and MD participated in the analysis and interpretation of data; NA, MA, MAM, PA, and KBL were involved in drafting and revising the manuscript. All authors read and approved the final manuscript.

## Supplementary Material

Additional file 1 **Appendix 1**. Click here for file
