# Supplementary material for: Assessment of scientific thinking in basic science in the Iranian second national Olympiad
Source: BMC Res Notes. 2012 Jan 23;5:61. doi: 10.1186/1756-0500-5-61 (PMC3292993; doi:10.1186/1756-0500-5-61)
Supplement: Additional file 1 — Appendix 1. [file 1756-0500-5-61-S1.DOC]

Exam I: Drawing a concept map

*Dear Participants:*

In this section

A) You should draw a concept map about differentiation of embryonic stem cells to mature nervous system cells. Please notice that you should draw the best concept map that can define possible mechanism of this differentiation based on the three research articles and one page explaining the mechanism that is in the enclosed pages

B) Explain the relationships in your concept map in one A4 page

*Enclosed page:*

Differentiation of embryonic stem cells to mature nervous system cells nowadays is an important for treatment of end stage diseases. As you know, the premature neuronal cells in central nervous system in embryonic period differentiate to oligodendrocyte, astrocytes and neurons.

Today with advancement in science, production of mature nervous system cells from stem cells is possible. Stem cells are divided in to two groups: embryonic stem cells and adult stem cells. Three ways are suggested to differentiate stem cells to mature cells:

1- Using specific growth factors

2-Differentiation of stem cells to ectoderm, mesoderm and endoderm (named as embryonic bodies) and maturation of these embryonic bodies

3-Direct differentiation by gene technology

In each of the above mentioned ways response is dependent to ligand, its receptors and time of exposure. One of the important way for getting the best response is change in "genes' expression proteins" and one of the best way is using micro RNA (miRNA). miRNAs are post-transcriptional regulators that bind to complementary sequences on target [**messenger RNA transcripts**](http://en.wikipedia.org/wiki/Messenger_RNA), usually resulting in translational repression or target degradation and [**gene silencing**](http://en.wikipedia.org/wiki/Gene_silencing).

*Exam II: Hypothesis generation*

A researcher inserted miRNA-124 gene by lentiviruse vector into stem cells. After differentiation of stem cells to mature neuronal cells he understood that the amount of miRNA 124 is reduced significantly. It should be mentioned that these viruses are expressing genes continuously. With respect to your drawn concept map, generate a hypothesis that define and explain the above mentioned mechanism.

Notice that you should write only one hypothesis

*Exam III: Choosing variables based on the hypothesis*

One of the suggested hypotheses about reduction of miRNA 124 is reduce to change pri-miRNA to mature miRNA. With respect to your drawn concept map and testing the above mentioned hypothesis which of the below variables should be chosen?

Please explain why you chose or didn't choose this variable.

1-Mature miRNA-124

2-pri - miRNA

3- Final protein product from miRNA 124

4-Phosphorylated and unphosphorylated STAT 3

5-Protein kinase enzyme

6-Dicer enzyme

7-Markers of neuronal cells differentiation

*Exam IV: Scientific thoughts measuring*

A researcher wants to improve mature neuronal cell production by co culture method. In this method he cultured the embryonic stem cells with brain nervous system cells as a feeder layer. This leads to increase neuronal differentiation. The hypothesis is:

*"Inhibition of the BMP4 molecule results to accelerate adult neuronal cell differentiation"*

Decide which of the following statements approve or reject the hypothesis:

|  | Reject (-) | Neutral value (0) | Approve (+) |
| --- | --- | --- | --- |
| 1- If dbcAMP is added, it leads to more differentiation toward neuronal precursor. |  |  |  |
| 2- If miRNA-124 presents in the feeder layer, it leads to increase differentiation of neuronal cells. |  |  |  |
| 3- If a membrane which inhibits contact of feeder layer with stem cells is used, then it leads to inhibit the neuronal cell differentiation. |  |  |  |
| 4- If using anti Noggin antibody, it leads to increased neuronal differentiation. |  |  |  |
| 5- If expressing protein BMP4 in feeder layer leads to increased neuronal differentiation. |  |  |  |
| 6- If adding bFGF to culture leads to increase neuronal differentiation. |  |  |  |
| 7- If exocytosis of Noggin is inhibited in the feeder cells; it leads to decrease neuronal differentiation. |  |  |  |
| 8- If adding anti Noggin Ab, it leads to decrease glial cells differentiation. |  |  |  |
| 9- If expressing siRNA against BMP4 gens in ESC cells, it leads to decrease mesodermal differentiation. |  |  |  |
| 10- If expression of Noggin protein increased in feeder cells, it leads to decrease neuronal differentiation. |  |  |  |
| 11- If expression of Noggin increases in feeder cells, it leads to increase nestin positive cells |  |  |  |
| 12- If a destructive mutation in BMP4 receptors is happen, it leads to increase neuronal cells differentiation |  |  |  |
